# Amyloid-β can activate JNK signalling via WNT5A-ROR2 to reduce synapse formation in Alzheimer's disease

**DOI:** 10.1242/jcs.263526

**Published:** 2025-02-05

**Authors:** Kevin Fang, Ehsan Pishva, Thomas Piers, Steffen Scholpp

**Affiliations:** ^1^ Living Systems Institute, University of Exeter, Exeter EX4 4QD, UK; ^2^ Department of Psychiatry and Neuropsychology, Mental Health and Neuroscience Research Institute, University Maastricht, 6229 ER Maastricht, The Netherlands; ^3^ University of Exeter Medical School, RILD Building, RD&E Hospital Wonford, Exeter EX2 5DW, UK

**Keywords:** Wnt/PCP, JNK signalling, Synaptogenesis, Alzheimer's disease, Presenilin1, KOLF2.1J

## Abstract

Wnt signalling is an essential signalling system in neurogenesis, with a crucial role in synaptic plasticity and neuronal survival, processes that are disrupted in Alzheimer's disease (AD). Within this network, the Wnt/β-catenin pathway has been studied for its neuroprotective role, and this is suppressed in AD. However, the involvement of the non-canonical Wnt-planar cell polarity (Wnt/PCP) pathway in AD remains to be determined.

This study investigates the role of ROR2, a Wnt/PCP co-receptor, in synaptogenesis. We demonstrate that WNT5A-ROR2 signalling activates the JNK pathway, leading to synapse loss in mature neurons. This effect mirrors the synaptotoxic actions of Aβ_1-42_ and DKK1, which are elevated in AD. Notably, blocking ROR2 and JNK mitigates Aβ_1-42_ and DKK1-induced synapse loss, suggesting their dependence on ROR2. In induced pluripotent stem cell (iPSC)-derived cortical neurons carrying a PSEN1 mutation, known to increase the Aβ_42/40_ ratio, we observed increased WNT5A-ROR2 clustering and reduced numbers of synapses. Inhibiting ROR2 or JNK partially rescued synaptogenesis in these neurons. These findings suggest that, unlike the Wnt/β-catenin pathway, the Wnt/PCP-ROR2 signalling pathway can operate in a feedback loop with Aβ_1-42_ to enhance JNK signalling and contribute to synapse loss in AD.

## Teaser

**Summary:** The non-canonical Wnt/PCP pathway, specifically the ROR2 receptor, has a role in Alzheimer's disease mediated by Aβ-induced synaptic dysfunction by activating JNK signalling.

## INTRODUCTION

Alzheimer's disease (AD) represents one of the most prevalent neurodegenerative disorders, afflicting millions worldwide with a profound impact on memory, cognition and overall quality of life (Lane et al., 2018). AD is characterised by chronic neuroinflammation, synaptic dysfunction, loss of neurons and brain atrophy. At the molecular level, the accumulation of amyloid-β (Aβ) peptides and tau protein tangles in neurons are some hallmark characteristics of AD, contributing significantly to the disease's pathogenesis (Hardy and Selkoe, 2002; Karran et al., 2011; Pluta et al., 2021; Tolar et al., 2020; Vogel et al., 2020; Wei et al., 2021). However, the intricate mechanisms underlying synaptic dysfunction and subsequent neurodegeneration remain incompletely understood, hindering the development of effective therapeutics.

Within this complex disease landscape, the Wnt signalling network emerges as a crucial regulator, offering insights into potential therapeutic targets for AD (Inestrosa and Arenas, 2010). Recent studies have highlighted a crucial role of the Wnt/β-catenin signalling pathway in the stabilisation of synaptic connections, neuronal survival and neurogenesis. In AD, these processes are disrupted, and indeed, Wnt/β-catenin signalling is down-regulated, and antagonists, such as DKK1 and DKK3, are upregulated (Flores et al., 2024; Inestrosa and Varela-Nallar, 2014; Purro et al., 2012). Among the Wnt signalling pathways, the non-canonical Wnt-planar cell polarity (Wnt/PCP) signalling, particularly WNT5A-mediated signalling, has gained emerging attention for its role in developing and maintaining the central nervous system (CNS; Chen et al., 2017). However, a link between dysfunctional Wnt/PCP signalling, CNS intensity and AD is unclear.

WNT5A, a ligand of the Wnt/PCP signalling pathway, regulates a wide range of cellular processes, including cell migration, polarity and adhesion, through mechanisms distinct from the canonical Wnt/β-catenin pathway (Sato et al., 2010). The function of WNT5A is complex and involves interactions with various co-receptors, including the receptor tyrosine kinase-like orphan receptors ROR1 and ROR2. For example, WNT5A-ROR2 signalling regulates the phosphorylation of Vangl2, which in turn facilitates the activation of the Wnt/PCP/Jun N-terminal kinase (Wnt/PCP/JNK) pathway. Interestingly, recent reports indicate that JNK signalling is upregulated in AD, has been linked to Aβ signalling (Shoji et al., 2000; Solas et al., 2023), and that JNK inhibitors have a neuroprotective function and increase neuronal survival (Harper and LoGrasso, 2001). The Wnt signalling network and its regulating influence on the JNK pathway could play a role in modulating neuronal survival. However, the involvement of WNT5A-ROR2 in regulating the effects of AD pathology and its interaction with Aβ signalling is less understood.

Given the intricate involvement of Wnt/PCP in neurogenesis and the potential impact of the JNK signalling pathway on AD progression, here, we assessed whether differential expression of known Wnt regulators was associated with AD amyloid pathology and identified WNT5A, ROR and JNK components. For our functional analysis, we used the human neuroblastoma cell line, SH-SY5Y treated with Aβ_1-42_ oligomers and compared the findings to what was seen in induced pluripotent stem cell (iPSC)-derived cortical neurons carrying the familial AD-linked PSEN1^A75V^ mutation, known to increase the plasma Aβ_42/40_ ratio, which is strongly linked to AD pathology (Hanon et al., 2018; Pantazis et al., 2022; Patel et al., 2019; Risacher et al., 2019). In SH-SY5Y-derived neurons, we observed that increasing WNT5A-ROR2 signalling led to the activation of the JNK signalling pathway and enhanced sprouting of protrusions along neurites. Simultaneously, we also observed a reduction in the clustering of pre- and post-synaptic markers, suggesting a decrease in synaptogenesis. This phenotype resembles that of SH-SY5Y-derived neurons treated with Aβ_1-42_ peptides, together resembling some key aspects of AD. Surprisingly, inhibition of WNT5A-ROR2/JNK signalling mitigates the phenotypes observed after Aβ_1-42_ and DKK1 treatment. In iPSC-derived cortical neurons carrying the PSEN1^A75V^ mutation, we found upregulation of WNT5A-ROR2 clusters and enhanced JNK signalling. Supporting our results in the SH-SY5Y neurons, blockage of JNK signalling in iPSC-derived cortical neurons harbouring the PSEN1^A75V^ mutation could mitigate the loss of synaptic cluster formation. Our results suggest that in AD, an imbalance in the Wnt signalling network – here, an increased Wnt/PCP signalling – is linked to Aβ signalling in a positive feedback loop reinforcing JNK signalling and hindering synapse formation.

## RESULTS

### Expression of Wnt/PCP components is upregulated in an AD post-mortem brain

**Fig. 1.**
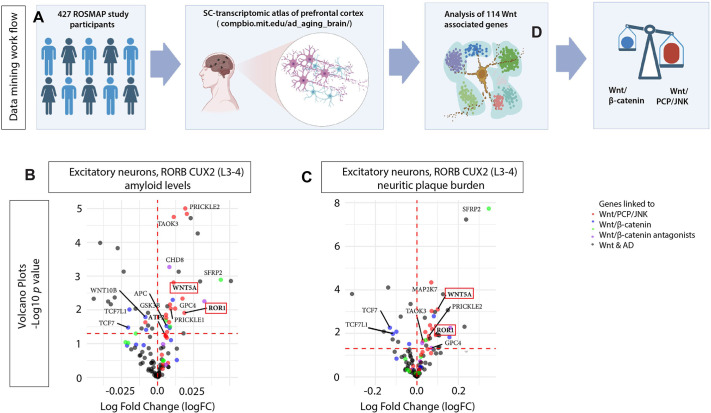
**Differential gene expression study for Wnt-related genes in AD.** (A) Cohort and snRNA-sequence profiling summary from 427 ROSMAP study participants along a spectrum of AD progression (based on Mathys et al., 2023). Created in BioRender by Scholpp, S., 2025. https://BioRender.com/j07k138. This figure was sublicensed under CC-BY 4.0 terms. (B,C) Volcano plots of the expression of 114 Wnt-related genes in excitatory neurons, RORB CUX2 of the layers 3 and 4 associated with amyloid levels (B), neuritic and diffuse plaque burden (C). The colour code indicates association with Wnt/PCP/JNK (red), Wnt/β-catenin (blue), Wnt/β-catenin antagonists (green) and AD-related genes (purple). The horizontal dashed line corresponds to *P*<0.05 (unpaired two-sided Student's *t*-test).

Although the pathological hallmarks of AD include amyloid plaque deposition and Tau protein hyperphosphorylation, the underlying molecular mechanisms remain incompletely understood. A recent landmark study establishing a comprehensive single-cell atlas of the human prefrontal cortex across 427 individuals reported transcriptional differences associated with multiple measures of AD pathology and identified AD-associated alterations conserved across cortical layers and neuronal subtypes (Mathys et al., 2023; Fig. 1A). We used this publicly available dataset to investigate the association of the Wnt signalling network in AD. Specifically, we focussed our analysis on the transcriptional profile of the excitatory cortical neurons of layers 3 to 4, which are affected before the onset of clinical symptoms (Jacobsen et al., 2006; Mairet-Coello et al., 2013). This neuronal population harbours crucial functions essential for memory and information processing and allows for a targeted exploration of Wnt signalling alterations potentially linked to early cognitive decline. In our analysis, we focussed on the differential expression of 114 genes from the Wnt network, focusing on ligands, receptors and downstream effectors to elucidate the specific pathways potentially driving the disease process (Fig. 1A; Table S1) associated with increasing amyloid concentration and plaque burden (Fig. 1B,C), we observed an imbalance between Wnt/β-catenin signalling and Wnt/PCP/JNK signalling in the excitatory neurons in layers 3 and 4. For example, we found an upregulation of some critical components of the Wnt/PCP/JNK pathway, like the ligands WNT5A and WNT5B, the cognate Wnt/PCP co-receptors of the ROR family and the downstream signalling factors JUN and ATF family proteins. Consistent with this, some canonical Wnt transcription factors were downregulated, like TCF7, TCF7L1 and LEF1. In parallel, known Wnt antagonists from the secreted frizzled-related protein (sFRP) and DKK family were upregulated. As a positive control, we also found a significant upregulation of genes associated with Wnt and AD, such as those encoding CHD8 and members of the CSNK1 family. These findings suggest an increase of the non-canonical Wnt/PCP/JNK signalling pathway in excitatory cortical neurons in AD, potentially impacting neuronal health and function.

### Expression of WNT5A, WNT5B and ROR2 is enhanced in Aβ-treated SH-SY5Y cells

**Fig. 2.**
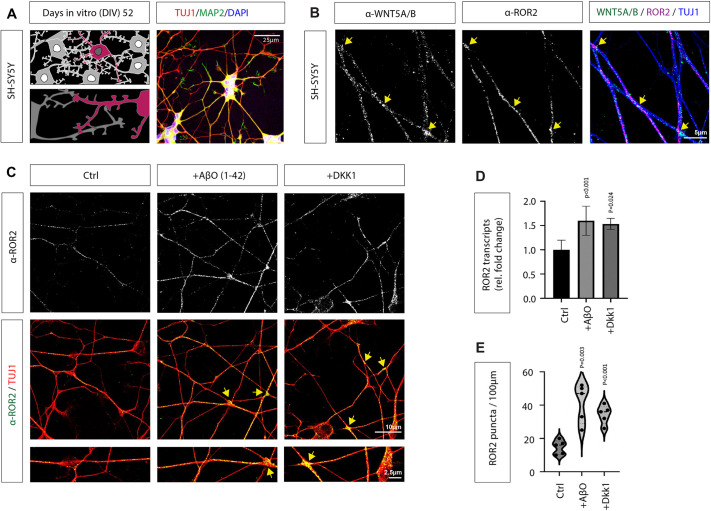
**The cognate receptor ROR2 is colocalised with the Wnt ligands WNT5A/B on dendrites of SH-SY5Y neuronal cells and is increased in an AD context.** (A) Schematic drawings of the morphology of SH-SY5Y cells as they differentiate into neuron-like cells over 52 days (top) with a focus on the forming neurite network (bottom). These cells were then characterised by TUJ-1 (red), MAP2 (green) and DAPI (blue). Scale bar: 25 µm. (B) IHC co-staining of mature SH-SH5Y neurons to map the expression of ROR2 and WNT5A/B. ROR2 is localised on neural dendrites and shows a robust colocalisation with WNT5A/B (yellow arrows). Scale bar: 5 μm. (C) IHC shows endogenous ROR2 expression after treatment with indicated compounds in SH-SH5Y neurons. Scale bars: 10 µm (main images); 2.5 µm (magnifications below main images). Images in A–C are representative of three repeats. (D) Quantification (mean±s.d.) of *ROR2* mRNA transcripts using *GAPDH* as a housekeeping gene for normalisation (E) and quantification (violin plot with median and quartiles indicated) of ROR2 protein puncta along neurites (100 µm) demonstrates the upregulation of ROR2 after treatment with Aβ and DKK1. qRT-PCR was repeated twice. Six dendrites of each sample were selected to quantify ROR2 puncta and data from three biological replicates are displayed. *P*-values were calculated with an unpaired two-sided Student's *t*-test.

To determine whether there is an effect of Wnt/PCP/JNK signalling in an AD context *in vitro*, we next established an SH-SY5Y-derived neuronal cell culture system. SH-SY5Y cells were plated on poly-D-lysine- and laminin-coated culture dishes in serum-free medium supplemented with nerve growth factor (NGF) and brain-derived neurotrophic factor (BDNF) to promote neurite outgrowth. After an initial proliferation period of 7 days, retinoic acid (RA) was introduced to the medium to induce terminal differentiation. An extended differentiation period of up to 60 days then allowed for the formation of extensive neuronal networks and expression of mature neuronal markers (Fig. 2A). After DIV 52, we found expression of class III β-tubulin (TUJ-1) at high levels throughout the entire neuron, including dendrites, axons, and the cell body and microtubule-associated protein 2 (MAP2) primarily localised in the dendrites and cell body of mature neurons. The differentiated and mature SH-SY5Y neurons were then used to determine the expression of the Wnt/PCP/JNK key regulators, WNT5A and WNT5B (antibody recognises both proteins, and is denoted anti-WNT5A/B) and ROR2. In our immunohistochemistry (IHC) experiments, we found localisation of WNT5A/B and ROR2 individually and together in prominent clusters predominantly localised to crossing neurites (yellow arrows) (Fig. 2B; antibody controls with anti-ROR2 antibody are in Fig. S1A,C and with anti-WNT5A/B antibody in Fig. S1B,D). To mimic an AD background chemically, we treated the neurons with 4 µM Aβ oligomers (Aβ_1-42_) or 200 ng/ml DKK1 protein for 24 h. We found that *ROR2* mRNA expression, as well as ROR2 protein localisation on neurites, is significantly increased in SH-SY5Y neurons treated with AβO_1-42_ and DKK1 (Fig. 2C–E). The number of *ROR2* mRNA transcripts increased ∼1.60 and 1.53-fold, whereas the fold change for ROR2 protein puncta was 2.82 and 2.32, respectively.

### Soluble AβO can activate the Wnt/PCP/JNK pathway in a ROR2-dependent manner

**Fig. 3.**
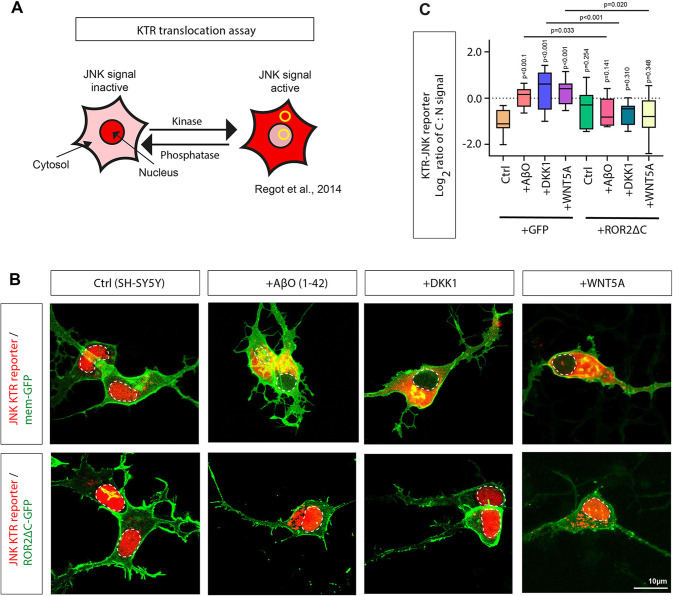
**ROR2 facilitates JNK signalling in SH-SY5Y neurons in AD conditions.** (A) Schematic representation of the functioning of the KTR–mCherry reporter. The reporter is localised to the nucleus at low JNK activity; when JNK signalling is activated, phosphorylated JNK–KTR mCherry translocates to the cytoplasm. The signal ratio of cytoplasm:nucleus (C:N) is used to quantify JNK signalling activation. (B) Live images of SH-SY5Y neurons transfected with JNK-KTR–mCherry and Gap43–GFP or ROR2-ΔCRD. Soluble AβO_1-42_, DKK1 and WNT5A protein were added at 24 h prior to imaging. The white dashed line indicates the nucleus. (C) The C:N ratio of 8 cells of three biological repeats were calculated. The box represents the 25–75th percentiles, and the median is indicated. The whiskers show the minimum to maximum value. (C:N mean values: ctrl, 0.49; +Aβ, 0.98, +DKK1, 1.00; +WNT-5A; 0.80; ROR2-ΔCRD, 0.57; ROR2-ΔCRD+Aβ, 0.59; ROR2-ΔCRD+DKK1, 0.75; ROR2-ΔCRD+WNT-5A, 0.69). *P*-values were calculated with a one-way ANOVA with Dunnett post test where indicated.

WNT5A and ROR2 are crucial components of the Wnt/PCP/JNK signalling pathway. Therefore, we tested whether treating SH-SY5Y neurons with AβO_1-42_ and DKK1 influences JNK signalling. Thus, we used a JNK signalling reporter system to quantify the Wnt/PCP response in receiving neurons. Specifically, we used the JNK kinase translocation reporter, JNK KTR–mCherry, to monitor alteration in the JNK signalling cascade (Regot et al., 2014). JNK-KTR–mCherry localises to the nucleus (N) in its dephosphorylated state (low JNK activity, Fig. 3A). Upon activation by phosphorylation, it shuttles to the cytoplasm (C) within minutes. To decipher the response of SH-SY5Y neurons to AβO_1-42_ and DKK1 transfected with the JNK-KTR–mCherry, the C:N ratio was calculated as an indicator of JNK signalling strength in near real-time (Zhang et al., 2024). SH-SY5Y neurons treated with AβO_1-42_ and DKK1 showed translocation of KTR into the cytoplasm, and the C:N ratio was significantly upregulated. AβO_1-42_-treated neurons showed a 3.02-fold increased C:N ratio, and DKK1-treated cells showed a 3.04-fold increase, indicating an activation of the JNK signalling pathway (Fig. 3B,C). As a positive control, we treated the neurons with WNT5A protein, which led to a 2.65-fold increase, similar activation of the ROR2/JNK signalling indicated by the translocation of the KTR reporter.

Next, we asked whether the activation of the JNK signalling pathway depends on the Wnt/PCP receptor ROR2. Therefore, we co-transfected a dominant-negative ROR2 construct lacking the intracellular kinase domain (ROR2-ΔC) together with the KTR reporter. We observed that AβO_1-42_, DKK1 and WNT5A cannot activate the JNK signalling significantly if the ROR2 function is blocked (Fig. 3B,C). JNK signalling facilitates filopodia formation (Brunt et al., 2021), and in support of our findings, we found that AβO_1-42_- and DKK1-treated neurons or neurons transfected with ROR2 form more dendritic filopodia, whereas expression of ROR2-ΔC led to a significant downregulation of the filopodia density on neurites (Fig. S2). These results suggest that, in an AD context, when AβO_1-42_ and DKK1 are upregulated, neurons can activate JNK signalling. However, activation of JNK signalling is facilitated by functional ROR2 receptors.

**Fig. 4.**
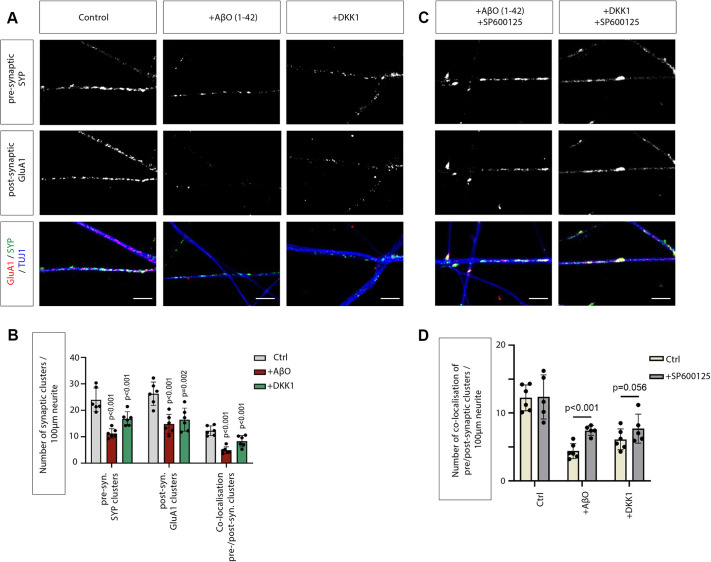
**Treatment of SH-SY5Y neurons with AβO_1-42_ and DKK1 reduces synaptic marker localisation, which can be alleviated by blockage of JNK signalling.** (A) Confocal images of SH-SY5Y neurons labelled with the presynaptic marker synaptophysin (SYP, green), the postsynaptic glutamate receptor (GluA1, red) and β-tubulin (blue). Colocalisation of SYP with GluA1 indicates possible synaptic connections between SH-SY5Y neuron cells. Scale bars: 5 μm. (B) Quantification of number of synaptic clusters for results in A. (C) Confocal images show the clusters of synaptic markers pre-treated with JNK inhibitor SP600125. Quantification of the number of colocalisation of synaptic clusters is shown in D. (B,D) In the quantifications, five dendrites of each sample were selected to quantify the number of puncta, and six samples of three biological replicates are displayed. Error bars are mean±s.d. *P*-values were calculated with an unpaired two-sided Student's *t*-test.

Next, we wanted to understand the consequences of upregulated Wnt/PCP/JNK signalling on synaptogenesis. Therefore, we treated SH-SY5Y neurons with AβO_1-42_ and DKK1 and investigated the expression of the presynaptic marker synaptophysin (SYP) and the postsynaptic marker ionotropic glutamate receptor, AMPA1 (GluA1, encoded by *GRIA1*). We found that the number of clusters of SYP and GluA1 on neurites is significantly reduced upon treatment with AβO_1-42_ and DKK1 (Fig. 4A,B). Similarly, we found a significant reduction of clusters with colocalised SYP and GluA1, suggesting decreased synaptogenesis. We then asked whether the observed phenotype depends on the activation of the Wnt/PCP/JNK pathway. To assess the role of JNK signalling in this process, we treated SH-SY5Y neurons with AβO_1-42_ or DKK1 and subsequently with a small-molecule inhibitor of JNK kinase activity. SP600125 specifically inhibits all three kinases JNK1–JNK3 (also known as MAPK8–MAPK10) within minutes without inhibition of ERK1 or ERK2 (MAPK3 and MAPK1), phospho-p38 MAPKs (MAPK11–MAPK14) or ATF2 (Bennett et al., 2001). Blockage of JNK signalling did not alter the number of clusters with colocalised SYP and GluA1 in control neurons. However, if pre-treated with AβO_1-42_ and DKK1, SP600125 treatment led to a significant increase in the emergence of colocalised SYP and GluA1 clusters in SH-SY5Y neurons (Fig. 4C,D). These data suggest that the downregulation of colocalised SYP and GluA1 clusters in SH-SY5Y neurons by AβO_1-42_ and DKK1 depends on JNK signal activation.

### The Wnt/JNK receptor ROR2 expression depends on PSEN1 function

**Fig. 5.**
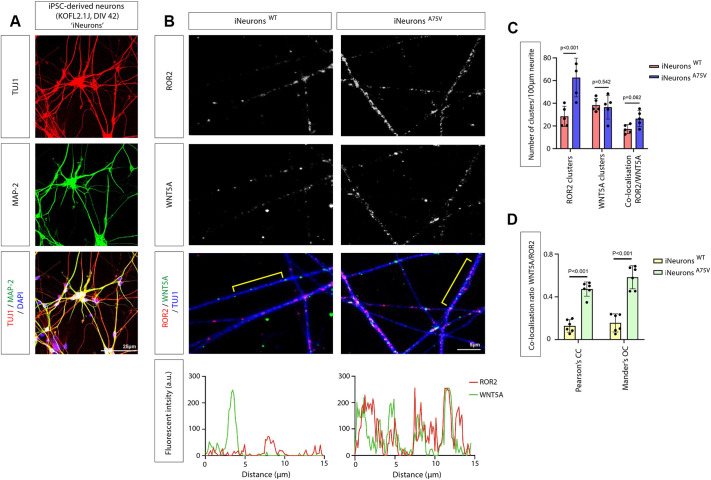
**WNT5A/B and ROR2 expression in iPSC-derived cortical iNeurons.** (A) iPSC-derived KOLF2.1J cortical neurons, called iNeurons, after 42 days *in vitro* (DIV 42), show a robust expression of TUJ-1 (red) and MAP2 (green). DAPI staining is in blue. Scale bar: 25 µm. (B) IHC staining of WT iNeurons and iNeurons carrying the PSEN1^A75V^ mutation indicates the colocalisation of ROR2 and WNT5A/B. Scale bar: 5 µm. Yellow lines indicate an example of fluorescent intensity measurement of WNT5A and ROR2 along a 15 µm area of a neurite displayed as graphs. (C,D) Quantifications for number of clusters (C) and colocalisation levels (D). For D, the comparative analysis of colocalisation of WNT5A and ROR2 with Pearson correlation coefficient (PCC) and Manders’ overlap coefficient (MOC), respectively. In the quantifications, five dendrites of each sample were selected to quantify puncta and intensity, and six samples of three biological replicates are displayed. Error bars are mean±s.d. *P*-values were calculated with an unpaired two-sided Student's *t*-test. a.u., arbitrary units.

To complement the findings from the SH-SY5Y cells in a more pathophysiological context, we established iPSC-derived cortical neuron cultures (denoted ‘iNeurons’; Piers et al., 2024). From the iNDI project, we obtained the parental KOLF2.1J iPSC line and genome-edited daughter lines carrying the familial AD (fAD)-linked mutation PSEN1^A75V^ (Ramos et al., 2021). Comparing the isogenic cell lines provides a unique and powerful tool for dissecting the role of specific fAD-linked mutations known to increase the pathological AβO_42/40_ ratio. Here, we investigate the PSEN1^A75V^ mutation, which increases the Aβ_42/40_ ratio in mice and is associated with early-onset AD (Kauwe et al., 2007; Sun et al., 2017). After differentiation over 42 days *in vitro* (DIV), we found the expression of TUJ-1 and MAP2, indicating a ‘young’ cortical neuron phenotype (Fig. 5A). We used this neuronal cell culture system to compare the expression of ROR2 and WNT5A between wild-type (WT) iNeurons and PSEN1^A75V^ iNeurons. We observed that the number of ROR2 clusters is increased 2.18-fold along neurites of neurons carrying this PSEN1 mutation, which consequently led to a 1.52-fold increase in WNT5A and ROR2 co-clusters (Fig. 5B,C). Similarly, the colocalisation for WNT5A with ROR2 along the neurites increased significantly (Fig. 5D). These data suggest that an increase in the AβO_42/40_ ratio can induce an increase in ROR2 expression in human iNeurons. Interestingly, we found no alteration in WNT5A clusters, suggesting that the production of the ligand is unaltered.

**Fig. 6.**
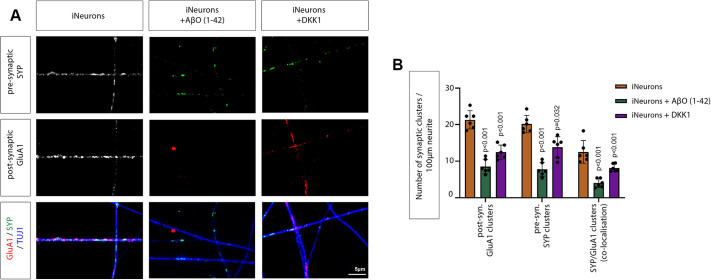
**Treatment of soluble AβO_1-42_ and DKK1 affects synaptic clusters in iNeurons.** (A) Confocal imaging of WT iNeurons IHC labelled for the presynaptic marker SYP (green), the postsynaptic marker GluA1 (red) and β-tubulin (blue). Colocalisation of SYP with GluA1 shows potential synaptic connections. Scale bar: 5 μm. (B) Quantifications for number of clusters for iNeurons as in A. In the quantification, five dendrites of each sample were selected to quantify puncta and six samples of three biological replicates are displayed. Error bars are mean±s.d. *P*-values were calculated with an unpaired two-sided Student's *t*-test. Shared controls were used for the simultaneously performed experiments shown in this figure and Fig. 7.

Next, we investigated the effect of AβO_1-42_ and DKK1 treatment on the clustering of pre- and post-synaptic markers in iNeurons. We found that treating iNeurons with AβO_1-42_ or DKK1 significantly reduced the formation of pre-synaptic SYP clusters by 1.61-fold and 1.42-fold, respectively (Fig. 6A,B). The post-synaptic GluA1 clusters were similarly decreased, here 1.60-fold and 1.39-fold, respectively. Consequently, the formation of colocalised SYP and GluA1 co-clusters was also reduced by 1.70-fold and 1.36-fold (Fig. 6A,B), in accordance with our previous observation in SH-SY5Y-derived neurons. Together with the previous experiment, these data suggest that AD-linked mutation PSEN1^A75V^ in KOLF2.1J-derived iNeurons might lead to an increase in ROR2 expression and, subsequently, a decrease of pre- and post-synaptic clusters.

**Fig. 7.**
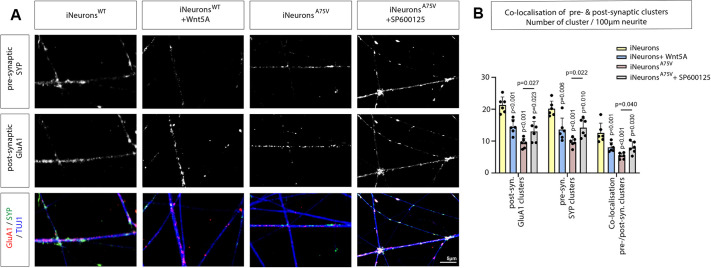
**JNK inhibitor alleviates synaptic loss in PSEN1^A75V^ iNeurons.** (A) Confocal imaging of iNeurons labelled with the presynaptic marker SYP (green), the postsynaptic marker GluA1 (red) and β-tubulin (blue). Colocalisation of SYP with GluA1 shows potential synaptic connections after treatment with the indicated compounds. Scale bar: 5 μm. (B) Quantifications for number of clusters for iNeurons as in A. In the quantification, five dendrites of each sample were selected to quantify puncta and six samples of three biological replicates are displayed. Error bars are mean±s.d. *P*-values were calculated with including a one-way ANOVA test with Dunnett post test. Shared controls were used for the simultaneously performed experiments shown in this figure and Fig. 6.

Based on our gene association analysis and data from the SH-SY5Y system, we hypothesise that Wnt/PCP/JNK signal activation reduces synaptic connections. Therefore, we treated WT iNeurons with WNT5A protein and found that the number of pre- and post-synaptic clusters was significantly decreased, here by 1.38-fold (Fig. 7A,B). Finally, we wondered whether PSEN1^A75V^ iNeurons show a reduced ability to cluster synaptic markers because of elevated Wnt/PCP/JNK signalling levels. Therefore, we treated the neurons with the JNK inhibitor SP600125 and found that we could partially rescue the pre- and post-synaptic puncta loss (Fig. 7A). We observed a significant 1.48-fold increase in puncta after blocking JNK signalling (Fig. 7B). Taking these findings together, these data suggest a central role for Wnt/PCP/JNK signalling in synaptic integrity and an increase of Wnt/PCP/JNK in an AD context.

## DISCUSSION

AD is a progressive neurodegenerative disorder characterised by the accumulation of Aβ plaques and neurofibrillary Tau tangles, leading to neuronal loss and cognitive decline. In parallel, several major signalling pathways are misregulated during various stages of AD progression. For example, the canonical Wnt signalling pathway is suppressed in AD, whereas their cognate inhibitors, like DKK1–DKK3, are massively upregulated (Nusse and Clevers, 2017). These findings accelerated the research on the first specific Wnt agonist for the carboxylesterase Notum in AD (Zhao et al., 2021). Emerging evidence also highlights a critical role for the non-canonical Wnt signalling pathway in AD, which is regulated by WNT5A. WNT5A can initiate a cascade of events leading to the activation of the small GTPases RHO, RAC and CDC42. In turn, this activates RHO kinases (ROCKs) and JNK signalling. This cascade is crucial for various cellular processes in development and tissue homeostasis, including cell migration, differentiation and apoptosis. However, how this signalling pathway is altered in AD and what the consequences are on neuronal function and health is unclear.

### An upregulation of Wnt/PCP/JNK is associated with AD

Here, we examined the association between expression of Wnt signalling network genes and AD at single-cell resolution (Mathys et al., 2023). This transcriptomic atlas of the aged human prefrontal cortex contains data from 427 ROSMAP study participants with varying degrees of Alzheimer's disease progression, cognitive impairment and AD pathology. Specifically, we analysed the expression for 114 Wnt/β-catenin and Wnt/PCP/JNK-related genes (Table S1) in excitatory neurons around the cortical layers 3–4. We then selected the variables for plaque burden and amyloid levels and found an enrichment of genes related to the Wnt/PCP/JNK signalling system. In contrast, genes associated with the Wnt/β-catenin pathway were reduced.

In the subsequent analysis, we investigate the effect of Aβ signalling on young neurons, which complicates the direct translational ability of the findings. Based on the finding that WNT5A/PCP/JNK is upregulated, we selected WNT5A, together with its cognate co-receptor ROR2, as a crucial signalling regulator for JNK signalling in AD for the subsequent analysis, as the ROR genes show redundancy in synaptogenesis (Ramos et al., 2021).

### ROR proteins are localised on neurites and activate Wnt/PCP signalling

To study their function in an AD context, we compared the effect of AβO in neuron-like cells differentiated from SH-SY5Y cells and iPSC-derived cortical neurons (iNeurons). Although SH-SY5Y cells offer a readily available and relatively easy-to-maintain *in vitro* model, their limitations make iPSC-derived neurons a more robust and versatile tool for studying complex neuronal functions and diseases. Specifically, SH-SY5Y cells are a cancerous human neuroblastoma cell line that can exhibit an immature dopaminergic neuronal phenotype. Therefore, we complemented our analysis with iPSC-derived cortical neurons, which were genetically modified to introduce specific mutations, thus creating a more controlled model for studying disease mechanisms. Here, we established the KOLF2.1J iPSC line from the iNDI project (Ramos et al., 2021). These iNeurons are derived from a single genetic background, increasing the linkage of a specific mutation to a phenotype rather than being a result of inherent genetic variations between cell lines. This is particularly important in AD research, where genetic predisposition plays a significant role. Here, we used a cell line harbouring a fAD variant linked to the A75V mutation in PSEN1, which is linked to early-onset AD and compared the effects to its isogenic control. Using these cell systems, we found that ROR2 expression is increased along neurites in an AD context.

Indeed, ROR2 is expressed in various neuronal populations, including in the hippocampus (Lein et al., 2007). In cultured hippocampal murine neurons, ROR1 and ROR2 have been detected during the neurite extension and synapse formation (Paganoni and Ferreira, 2003). Both ROR proteins are initially localised on MAP2-positive dendrites and have been suggested to regulate their outgrowth. For example, murine hippocampal neurons, treated with antisense oligonucleotides or siRNAs targeting *ROR1* or *ROR2*, extended shorter minor processes with fewer branching points. However, inhibition of ROR1 in hippocampal neurons can reduce synaptogenesis (Paganoni et al., 2010). The underlying mechanisms in regulating neurite extension in cortical neurons remain elusive.

### AβO shifts the balance between Wnt/β-catenin and Wnt/PCP signalling

Here, we show that the WNT5A and/or WNT5B can activate ROR2/PCP signalling in SH-SY5Y neurons and iPSC-derived human cortical neurons. Our data further suggest that ROR2 and the Wnt/PCP pathway can also be synergistically activated by high levels of AβO_1-42_ and DKK1, which have been observed in the AD context. The DKK1-mediated toxic effects of AβO_1-42_ could depend on the activation of the Wnt/PCP signalling pathway (Killick et al., 2014). In support of our findings, recent studies with rodent cortical neurons suggest that AβO induces Dkk1, which might shift Wnt signalling from the Wnt/β-catenin pathway towards the Wnt/PCP pathway, leading to a synapse loss and further AβO production (Sellers et al., 2018). Furthermore, inhibition of this Aβ–DKK1–Aβ positive feedback loop with Fasudil in primary rat cortical neuronal culture suggests a potential treatment for AD (Elliott et al., 2018). These results are further supported by the observation that KOLF2.1^A75V^-derived human cortical neurons also significantly increase membranous ROR2 expression. Consistently, we found that inhibition of ROR2 and the Wnt/PCP pathway can partially rescue the inhibitory effect of AβO_1-42_ and DKK1 signalling. These observations assign a key role for ROR2 and the Wnt/PCP pathway in Aβ signalling and subsequently in AD.

### Wnt/PCP/JNK signalling in AD

Finally, our data suggest that WNT5A and ROR2 are regulators of JNK signalling in the AD context. JNK signalling plays a significant role in regulating neuronal death, a hallmark of AD pathology. Previous studies have shown increased phosphorylated JNK (pJNK) expression in human postmortem brain samples from individuals with AD and a positive colocalisation with Aβ (Zhu et al., 2001). Similarly, we observed upregulation of the expression of JNK-related genes by differential expression analysis (Fig. 1). Upon activation, JNK phosphorylates various substrates, including the transcription factor c-Jun, leading to the induction of pro-apoptotic genes (Davis, 2000). The activation of JNK signalling has also been linked to the presence of AβO. It has been suggested that the interaction between AβO and neuronal receptors triggers a cascade of intracellular signals, culminating in the activation of JNK (Yan et al., 1996). Specifically, JNK3 is strongly enriched in the nervous system and involved in neurite formation and neurodegeneration (Mielke and Herdegen, 2000; Yarza et al., 2016). Phosphorylated JNK3 (pJNK3) is highly correlated with AD pathology, such as promoting Aβ plaques and causing neuroinflammation, leading to nerve cell apoptosis. The level of JNK3 in the cerebrospinal fluid (CSF) of individuals with AD positively correlates with cognitive decline (Gourmaud et al., 2015). A positive feedback loop regulates the formation of Aβ_1-42_: pJNK phosphorylates APP at Thr668 to promote the production of Aβ_1-42,_ and Aβ_1-42_ reacts on JNK to promote its phosphorylation (Musi et al., 2020). Finally, JNK signalling is involved in forming and aggregating neurofibrillary tangles (NFTs) (Pearson et al., 2006). Recent evidence suggests that inhibitors of JNK3 could be beneficial in AD (Qin et al., 2022). Here, we show that WNT5A-ROR2 is important for JNK signalling in cortical neurons. Blockage of ROR2 function or inhibition of JNK signalling can rescue the loss of pre- and post-synaptic marker localisation on neurites. In parallel, we found that increased levels of AβO also reduce pre- and postsynaptic marker localisation on neurites – again, this phenotype can be partially rescued by JNK inhibition. In parallel, AβO regulates GluA1 internalisation in HEK293T cells and primary cultured hippocampal and cerebrocortical neurons, suggesting an AβO-induced GluA1 suppression in neurons in early AD (Midorikawa et al., 2024). It is unclear whether JNK signalling is also involved in this process.

In conclusion, we provide evidence of an interconnected signalling network suggesting a potential mechanism by which non-canonical Wnt signalling influences AD progression in the cortex. The modulation of JNK signalling by WNT5A-ROR2, together with Aβ and DKK1, highlights the need to understand better the intertwined pathway interactions to develop targeted therapeutic strategies for AD.

## MATERIALS AND METHODS

### Plasmids and antibodies

The following plasmids were used in transfection experiments: pCS2+ Gap43-GFP (membrane-GFP); pCS2+-membrane-mCherry (Mattes et al., 2018); pCDNA-ROR2-GFP (Rogers et al., 2023), JNK KTR-mCherry (Regot et al., 2014) and ROR2ΔC-GFP (Rogers et al., 2023).

The following primary antibodies were used for immunofluorescence: anti-ROR2 (Santa Cruz Biotechnology, 1:200, H-1; Rogers et al., 2023), anti-WNT5A/B (Proteintech, 1:250, 55184-1-AP; Zhang et al., 2024), anti-GluA1 (Synaptic Systems, 1:250, 182 011), anti-synaptophysin 1 (Synaptic Systems, 1:250, 101 002), anti-β3-tubulin (Synaptic Systems, 1:500, 302 304) and anti-MAP2 (Merck, 1:500, MAB3418) antibodies. The following Alexa Fluor-conjugated (Thermo Fisher Scientific) secondary antibodies were used for immunofluorescence: donkey anti-mouse-IgG conjugated to Alexa Fluor 647 (abcam, 1:1000, ab150107), donkey anti-rabbit-IgG conjugated to Alexa Fluor 488 (abcam, 1:1000, ab150073) and goat anti-guinea pig-IgG conjugated to Alexa Fluor 568 (abcam, 1:1000, ab175714).

### qPCR

RNA for quantitative (q)PCR was collected from cell pellets using the QIAGEN RNeasy kit according to the manufacturer's instructions. qRT-PCR was then performed using the SensiFAST™ SYBR^®^ Lo-ROX One-Step Kit with half volumes according to the manufacturer's protocol and run using Applied Biosystems QuantStudio6 Flex. Primer sequences for ROR2 are forward 5′-ACTGGTCATCGCTTGCCTTT-3′ and reverse 5′-AGGCATGGAGACCTGTTTGT-3′. Data were normalised using *GAPDH* as a housekeeping gene, including standards in each qPCR run for both the genes, *ROR2* and *GAPDH*.

### Cell culture

#### SH-SH5Y neuroblastoma cells maintenance

SH-SY5Y neuroblastoma cells were obtained from Akshay Bhinge's laboratory (Living Systems Institute, University of Exeter, UK). Cells were maintained in DMEM/F12 (Gibco), supplemented with 10% FBS (Gibco). Lines were routinely passaged in a 1:5 split using 0.01% trypsin (Thermo Fisher Scientific). The cell line was tested regularly for mycoplasma by endpoint PCR testing every 3 months and broth tests every 12 months.

#### Differentiation of SH-SY5Y neuroblastoma into mature neurons

Neurons were derived as previously described (Shipley et al., 2016). Briefly, SH-SY5Y neuroblastoma cells were plated onto 0.01% poly-D-lysine- and laminin-coated six-well plates for starving culture by treating with DMEM/F12 plus 1% FBS supplemented with retinoic acid (10 μM; Merck). Medium was changed every other day from day (D)0–D8. On D8, cells were washed once with DMEM/F12 and cultures were replaced by neuronal differentiation medium [DMEM/F12 and Neurobasal medium (Thermo Fisher Scientific) in a 1:1 ratio, 10 mM HEPES, 1% N2 supplement, 1%B27 supplement and 1% Glutamax (Gibco)] supplemented with retinoic acid (10 μM) and BDNF (10 ng/ml; Peprotech). On forwards D8, medium was changed every other day and cells were maintained in culture until D40–60 for experimentation.

#### iPSC maintenance

iPSCs (KOLF2.1J) were obtained from the iNDI consortium (gift from Prof. Bill Skarnes, The Jackson Laboratory, Bar Harbor, USA). The KOLF2.1J*^A75V^* edited iPSC line used in this paper relates to dbSNP rs63749824, which has an alias in ClinVar (A79V). As such, cited research might annotate this mutation also as PSEN1^A79V^. Lines were maintained as colonies on human ES-qualified Matrigel (Corning) in StemFlex (StemCell Technologies). Colonies were routinely passaged in a 1:6 split using EDTA and banked. All cell lines were tested regularly for mycoplasma by endpoint PCR testing every 3 months and broth tests every 12 months.

#### Differentiation of iPSCs into cortical neurons

The differentiation process was described previously (Piers et al., 2024; Strano et al., 2020). Briefly, iPSCs were plated as colonies onto Matrigel and growing in neuronal differentiation medium [DMEM/F12 and Neurobasal medium in a 1:1 ratio, 10 mM HEPES, 1% N2 supplement, 1% B27 supplement, 1% Glutamax, 5 μM ascorbic acid and 20 μg/ml insulin (Thermo Fisher Scientific)] supplemented with SB431542 (10 μM; Tocris Bioscience) and LDN-193189 (0.2 μM; Tocris Bioscience) from D0-D12, replacing medium daily. On D12, cells were replated using accutase and growing in the same neuronal differentiation medium supplemented with bFGF (20 ng/ml; Peprotech), CHIR-99021 (1 μM; Tocris Bioscience) and Y-27632 (50 μM; Tocris Bioscience). On D13, medium was changed to neuronal differentiation medium supplemented with bFGF (20 ng/ml), CHIR-99021 (1 μM), and changed daily until D18. Cells can be banked and expanded neural progenitor cells (NPCs) on D18. For differentiating NPCs into cortical neurons, cells were maintained in differentiation medium supplemented with L-ascorbic acid (200 μM), BNDF (20 ng/ml), GDNF (10 ng/ml; Peprotech), and Compound E (0.1 μM; Biotechne) for 7 days before Compound E was removed. After D25, cultures were replenished every 3–4 days with a 50:50 medium change. At D40–60 cells were stained for cortical neuron lineage markers and used for experimentation.

### Neuronal transfection and treatment

Neuronal cultures were transfected with plasmid using a calcium-phosphate method as previously described (Hawkins et al., 2022; Piers et al., 2024). A plasmid-CaCl_2_ (12.4 mM) master mix was prepared for each combination to be transfected in Hank's balanced salt solution (HBSS). HBSS at the ratio of 1/8th of the DNA:CaCl_2_ mixture was added gradually to prevent the formation of large transfection complexes. The mixture was incubated for 15 min at room temperature (RT), then added to cultures and incubated at 37°C for 4.5 h, before a sodium acetate (300 mM in DMEM:F12) was added at 37°C for 5 min to dissolve precipitated complexes. Cultures were maintained in neuronal differentiation media supplemented for 24–48 h prior to imaging analyses.

### Pharmacological treatments of neuronal cultures

WNT5A and DKK1 human recombinant protein was reconstituted at 100 μg/ml in PBS and 0.1% BSA (Biotechne). Amyloid β (1-42) oligomers (Bachem, 4090148.05) were solubilised in DMSO at 1 mM, diluted to 100 mM in DMEM/F12, and allowed to oligomerise at RT for 16 h. 200 ng/ml WNT5A, 200 ng/ml DKK1 and 4 μM AβO_1-42_ were added and incubated at 37°C for 24 h before imaging or fixed for immunocytochemistry.

### Antibody staining and image acquisition

Cells were plated onto coverslips, and following indicated treatment or incubation, cells were immediately fixed using modified MEM-Fix (4% formaldehyde, 0.25–0.5% glutaraldehyde, 0.1 M Sorenson's phosphate buffer, pH 7.4) for 7 min at 4°C. Aldehydes were subsequently quenched by incubation with NaBH_4_ (0.1% w/v) for 7 min at RT. Further quenching was performed by three 10 min washes in PBS-glycine (0.2 M). Cells were then blocked and permeabilised in permeabilisation solution (0.1% Triton X-100, 5% donkey serum, 0.1 M glycine in 1× PBS) for 1 h at RT. Primary antibodies were diluted in incubation buffer (0.1% Tween 20, 5% serum in 1×PBS) and coverslips mounted on 40 μl spots overnight at 4°C in a humid environment. Coverslips were then washed with 1x PBS three times for 5 min before mounting on 40 μl spots of secondary antibodies diluted in incubation buffer for 1 h at RT. Coverslips were washed three times for 5 min with 1× PBS before mounting onto glass slides using ProLong Diamond Antifade mountant (Invitrogen) and left to dry overnight at 4°C before imaging.

### KTR–mCherry-based JNK reporter assay and analysis

SH-SY5Y neuron cells were transfected with JNK reporter KTR–mCherry and either/or Gap43–GFP and ROR2ΔC–GFP. For analysis, mean grey values were recorded for four regions of interest (ROIs) in the nucleus and four ROI in the cytoplasm per cell, and an average was taken. The ratio of cytoplasmic-to-nuclear signal was then recorded.

### Image acquisition and statistical analyses

Confocal microscopy images were performed on an inverted Leica TCS SP8 X laser-scanning microscope using a 63× water objective and analysed using ImageJ-FIJI software. For analysis of dendritic puncta, 3–5 neuronal dendrites within an ROI were selected randomly. The number of puncta was counted using the cell counter plugin in FIJI software, and an average number was taken. The number of colocalised puncta was quantified using the SynapcountJ plugin in FIJI software. Protrusion length was measured from the tip of the filopodia to the base, where it contacted the neurite. In the case of branching protrusions, one branch (the longest) would be measured. All experiments or conditions were repeated in biological triplicates. Statistical analyses were performed using GraphPad Prism version 10.1.2. Comparisons between the two groups were analysed using the unpaired two-sided Student's *t*-test.

### Post-mortem brain differential expression data acquisition

The association between Wnt pathway genes and overall amyloid levels, neuritic plaque burden, and diffuse plaque burden for excitatory neurons, specifically the RORB CLUX2 cell cluster (cortical layers 3–4), was obtained from the Mathys Lab GitHub repository (https://github.com/mathyslab7/ROSMAP_snRNAseq_PFC/tree/main/Results/Differential_gene_expression_analysis/). These results were generated using 427 post-mortem prefrontal cortex (PFC) samples from individuals with AD and individuals that did not have known dementia (as described by Mathys et al., 2023). The details of the single-cell RNA sequencing experiment and the differential expression analysis are described in Mathys et al., 2023. The Volcano plots were created using the ggplot2 R package (https://cran.r-project.org/web/packages/ggplot2; version 3.4.2).

## Supplementary Material

10.1242/joces.263526_sup1 Supplementary information
