# Supplementary material for: Amyloid-β can activate JNK signalling via WNT5A-ROR2 to reduce synapse formation in Alzheimer's disease
Source: J Cell Sci. 2025 Feb 5;138(3):JCS263526. doi: 10.1242/jcs.263526 (PMC11832185; doi:10.1242/jcs.263526)
Supplement: Supplementary information [file joces-138-263526-s1.pdf]

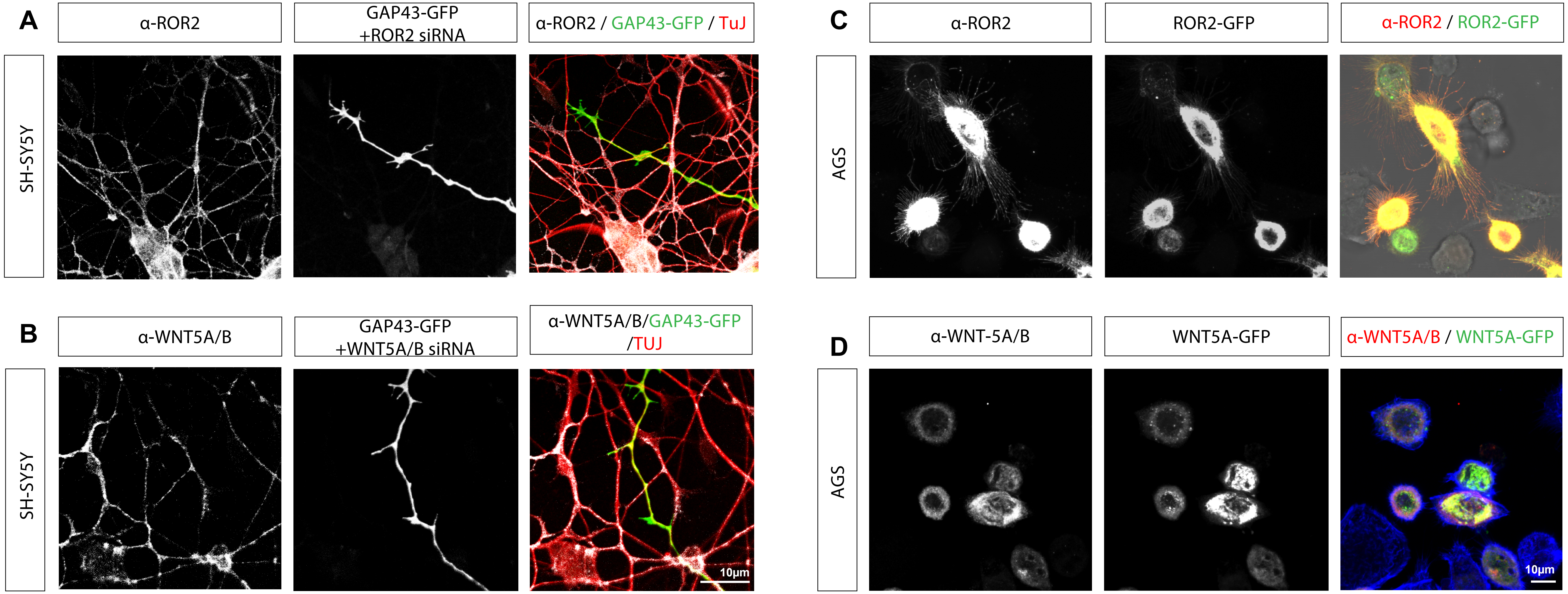

Fig. S1. Antibody specificity of ROR2 and WNT5A/B. (A) Confocal imaging of SHSY5Y neuron cells transfected with GAP43-GFP and ROR2 siRNA, cells were stained with αROR2 (green) and αTUJ1 (red). (B) Confocal imaging of SHSY5Y neuron cells transfected with GAP43-GFP and WNTA5/B siRNA, cells were stained with αWNT5A/B (green) and αTUJ1 (red). (C, D) Confocal imaging of AGS cells, which were transfected with ROR2-GFP or WNT5A-GFP and stained with αROR2 or αWN5A/B antibodies, respectively.

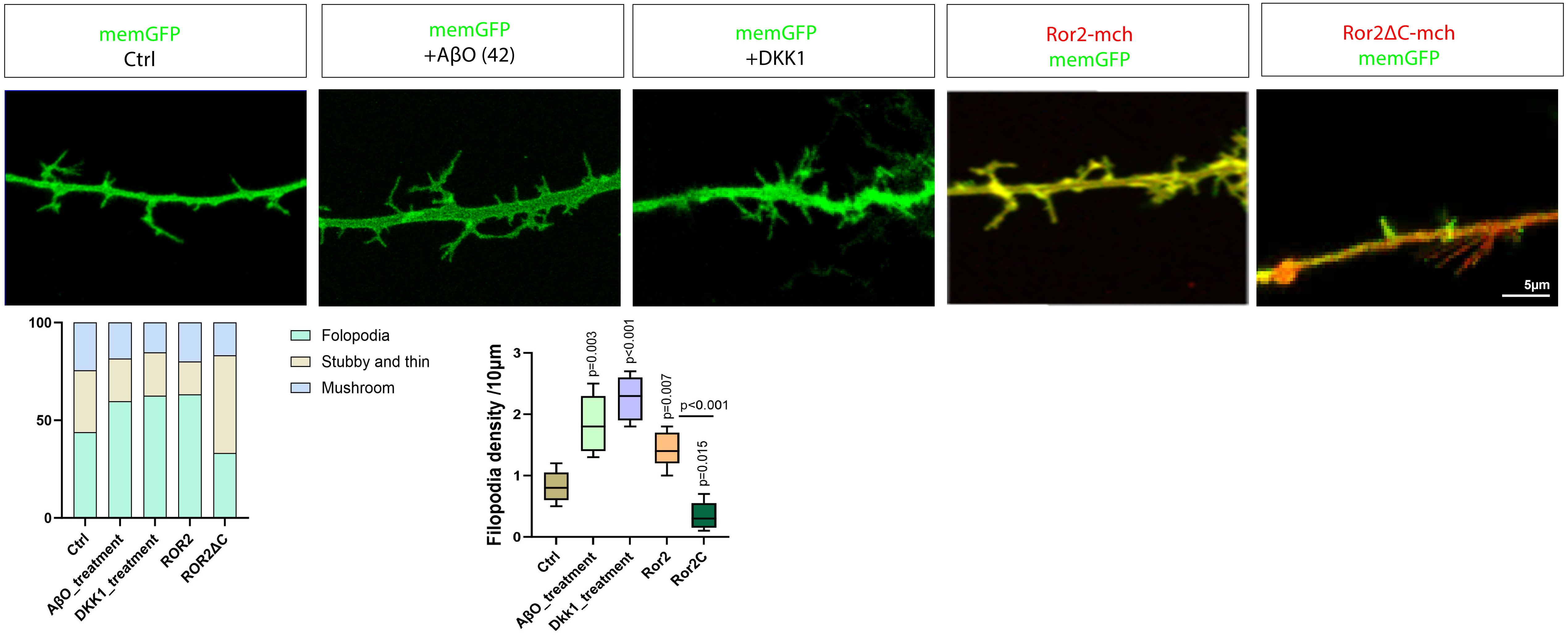

Fig. S2. Filopodia-like protrusion in AD context and is regulated by ROR2. (A) Confocal imaging of SHSY5Y neurons transfected with GAP43-GFP and transfected/treated with indicated markers/chemicals. (B) Percentage of filopodia, stubby, thin, and mushroom structure on the dendrite of SH-SY5Y neurons. (C) Density of filopodia-like protrusion on the dendrite of SH-SY5Y neurons. 5 dendrites of each sample were selected to quantify protrusions, and 6 samples of 3 biological replicates are displayed, including an unpaired Student-t-test with indicated *p* values.

Journal of Cell Science • Supplementary information

Table S1. Differential expression of 114 genes linked to Wnt signalling in the excitatory cortical neurons of layers 3 to 4 associated with increasing amyloid concentration and plaque burden based on (Mathys et al., 2023).

		Overall amyloid		Palque burden	
Gene_symbole	cluster_id	logFC	p_val	logFC	p_val
IGF1R	Exc L3-4 RORB CUX2	0.0192	9.90E-06	0.0714	9.45E-04
PRICKLE2	Exc L3-4 RORB CUX2	0.0203	1.44E-05	0.0584	1.33E-02
TAOK3	Exc L3-4 RORB CUX2	0.0113	1.77E-05	0.0300	2.21E-02
DKK2	Exc L3-4 RORB CUX2	0.0230	1.92E-05	0.0874	1.05E-03
CXXC4	Exc L3-4 RORB CUX2	0.0278	5.45E-05	0.1263	1.59E-04
GADD45A	Exc L3-4 RORB CUX2	-0.0398	1.04E-04	-0.1170	1.91E-02
RAC3	Exc L3-4 RORB CUX2	-0.0276	1.49E-04	-0.0396	2.44E-01
CHD8	Exc L3-4 RORB CUX2	0.0082	5.37E-04	0.0257	2.80E-02
WNT10A	Exc L3-4 RORB CUX2	-0.0235	7.37E-04	-0.1386	7.76E-05
WNT3	Exc L3-4 RORB CUX2	0.0146	7.39E-04	0.0455	3.65E-02
SFRP2	Exc L3-4 RORB CUX2	0.0439	1.28E-03	0.3468	1.86E-08
WNT7B	Exc L3-4 RORB CUX2	0.0509	1.39E-03	0.2297	4.99E-03
FZD1	Exc L3-4 RORB CUX2	0.0295	1.44E-03	0.2375	5.89E-08
MAP2K7	Exc L3-4 RORB CUX2	0.0111	1.55E-03	0.0697	4.51E-05
MECOM	Exc L3-4 RORB CUX2	-0.0297	4.29E-03	0.0069	8.83E-01
WNT5A	Exc L3-4 RORB CUX2	0.0173	4.61E-03	0.1008	7.98E-04
WNT6	Exc L3-4 RORB CUX2	-0.0443	4.67E-03	-0.3124	1.55E-04
CTNND2	Exc L3-4 RORB CUX2	0.0105	5.04E-03	0.0176	3.56E-01
CSNK1A1L	Exc L3-4 RORB CUX2	0.0325	5.58E-03	0.1614	5.62E-03
SFRP4	Exc L3-4 RORB CUX2	-0.0343	5.65E-03	-0.1596	8.32E-03
CCND1	Exc L3-4 RORB CUX2	-0.0324	6.84E-03	-0.1072	7.01E-02
CRK	Exc L3-4 RORB CUX2	0.0087	6.96E-03	0.0413	8.72E-03
BTRC	Exc L3-4 RORB CUX2	0.0068	7.80E-03	0.0089	4.86E-01
PRICKLE1	Exc L3-4 RORB CUX2	0.0091	9.12E-03	0.0130	4.64E-01
GPC4	Exc L3-4 RORB CUX2	0.0122	9.13E-03	0.0553	1.86E-02
HRAS	Exc L3-4 RORB CUX2	-0.0152	9.20E-03	-0.0123	6.63E-01
TCF7L1	Exc L3-4 RORB CUX2	-0.0195	9.73E-03	-0.0989	8.45E-03
SIAH1	Exc L3-4 RORB CUX2	-0.0066	1.22E-02	-0.0144	4.90E-01
ROR1	Exc L3-4 RORB CUX2	0.0186	1.23E-02	0.0920	1.20E-02
TAOK1	Exc L3-4 RORB CUX2	0.0058	1.39E-02	0.0070	5.54E-01
WNT10B	Exc L3-4 RORB CUX2	-0.0084	1.62E-02	-0.0375	3.10E-02
MAP2K4	Exc L3-4 RORB CUX2	0.0060	1.72E-02	0.0206	9.64E-02
GSK3B	Exc L3-4 RORB CUX2	0.0058	2.05E-02	0.0193	1.19E-01
RYK	Exc L3-4 RORB CUX2	0.0080	2.30E-02	0.0163	3.56E-01
MAP3K11	Exc L3-4 RORB CUX2	-0.0085	2.33E-02	-0.0181	3.15E-01
DVL3	Exc L3-4 RORB CUX2	-0.0066	2.91E-02	-0.0062	6.75E-01
APC	Exc L3-4 RORB CUX2	0.0063	3.01E-02	0.0095	5.05E-01
ZNRF3	Exc L3-4 RORB CUX2	0.0087	3.14E-02	0.0091	6.54E-01
TCF7	Exc L3-4 RORB CUX2	-0.0204	3.33E-02	-0.1304	5.52E-03
RSPO2	Exc L3-4 RORB CUX2	0.0084	3.44E-02	0.0186	3.52E-01
DUSP3	Exc L3-4 RORB CUX2	-0.0111	3.54E-02	0.0109	6.63E-01
CSNK2A1	Exc L3-4 RORB CUX2	-0.0038	3.56E-02	-0.0308	4.53E-04
PORCN	Exc L3-4 RORB CUX2	-0.0079	3.88E-02	-0.0051	7.85E-01
MAP3K5	Exc L3-4 RORB CUX2	0.0066	4.52E-02	0.0042	7.98E-01
FZD8	Exc L3-4 RORB CUX2	0.0180	4.95E-02	0.1495	8.63E-04
NKD2	Exc L3-4 RORB CUX2	-0.0151	5.02E-02	-0.0558	1.50E-01
ATF2	Exc L3-4 RORB CUX2	0.0055	5.87E-02	0.0181	2.09E-01
MAPK8IP3	Exc L3-4 RORB CUX2	0.0059	6.03E-02	0.0278	7.23E-02
RHOA	Exc L3-4 RORB CUX2	-0.0053	6.28E-02	-0.0035	7.98E-01
INVS	Exc L3-4 RORB CUX2	0.0064	6.69E-02	0.0065	7.07E-01
WNT2B	Exc L3-4 RORB CUX2	0.0093	7.88E-02	0.0819	1.65E-03
WIF1	Exc L3-4 RORB CUX2	-0.0222	9.03E-02	-0.0476	4.69E-01
NOTUM	Exc L3-4 RORB CUX2	-0.0204	9.52E-02	-0.0366	5.42E-01
CSNK2A2	Exc L3-4 RORB CUX2	0.0038	1.04E-01	0.0152	1.91E-01
WNT8A	Exc L3-4 RORB CUX2	-0.0149	1.07E-01	-0.1160	1.06E-02
WNT2	Exc L3-4 RORB CUX2	-0.0096	1.11E-01	-0.0219	4.61E-01
LEF1	Exc L3-4 RORB CUX2	-0.0212	1.13E-01	-0.0984	1.45E-01
FZD3	Exc L3-4 RORB CUX2	0.0040	1.27E-01	0.0387	1.93E-03
GADD45B	Exc L3-4 RORB CUX2	-0.0157	1.32E-01	0.0373	4.55E-01
FZD4	Exc L3-4 RORB CUX2	-0.0062	1.36E-01	-0.0325	1.08E-01
FBXW11	Exc L3-4 RORB CUX2	0.0024	1.37E-01	0.0084	2.93E-01
WNT7A	Exc L3-4 RORB CUX2	0.0134	1.40E-01	0.1078	1.24E-02
FLT4	Exc L3-4 RORB CUX2	-0.0183	1.47E-01	-0.0337	5.99E-01
RNF43	Exc L3-4 RORB CUX2	-0.0106	1.76E-01	-0.0904	2.46E-02
WNT9B	Exc L3-4 RORB CUX2	0.0074	1.93E-01	0.0417	2.27E-01
RASGRP1	Exc L3-4 RORB CUX2	-0.0049	2.01E-01	-0.0289	1.23E-01
DAAM1	Exc L3-4 RORB CUX2	0.0030	2.14E-01	-0.0039	7.42E-01
LRP5	Exc L3-4 RORB CUX2	-0.0118	2.19E-01	-0.0734	1.22E-01
DAAM2	Exc L3-4 RORB CUX2	-0.0124	2.26E-01	-0.0065	8.96E-01
JUN	Exc L3-4 RORB CUX2	-0.0089	2.63E-01	0.0148	6.98E-01
CTNNBIP1	Exc L3-4 RORB CUX2	0.0030	2.92E-01	-0.0002	9.87E-01
RSPO3	Exc L3-4 RORB CUX2	0.0134	3.06E-01	0.1573	1.51E-02
AXIN1	Exc L3-4 RORB CUX2	0.0042	3.15E-01	-0.0090	6.62E-01
AXIN2	Exc L3-4 RORB CUX2	0.0037	3.21E-01	0.0430	2.06E-02
APC2	Exc L3-4 RORB CUX2	-0.0034	3.31E-01	0.0203	2.20E-01
SFRP5	Exc L3-4 RORB CUX2	-0.0104	3.67E-01	-0.0106	8.58E-01
TCF7L2	Exc L3-4 RORB CUX2	-0.0041	3.71E-01	-0.0432	6.59E-02
MAP4K2	Exc L3-4 RORB CUX2	-0.0025	3.72E-01	-0.0033	8.14E-01
TRAF6	Exc L3-4 RORB CUX2	-0.0044	3.73E-01	-0.0783	1.72E-03
VANGL1	Exc L3-4 RORB CUX2	0.0072	3.74E-01	0.0689	8.13E-02
DKK1	Exc L3-4 RORB CUX2	-0.0117	3.98E-01	0.1150	7.97E-02
TAOK2	Exc L3-4 RORB CUX2	0.0039	4.02E-01	0.0639	4.29E-03
LRP6	Exc L3-4 RORB CUX2	0.0020	4.19E-01	-0.0011	9.26E-01
CSNK1E	Exc L3-4 RORB CUX2	0.0059	4.20E-01	0.0704	4.69E-02
TRAF2	Exc L3-4 RORB CUX2	0.0027	4.68E-01	-0.0361	5.51E-02
FZD6	Exc L3-4 RORB CUX2	0.0040	4.72E-01	0.0244	3.73E-01
EGFR	Exc L3-4 RORB CUX2	0.0075	4.87E-01	0.0932	8.32E-02
GADD45G	Exc L3-4 RORB CUX2	0.0075	4.94E-01	0.0627	2.55E-01
DVL1	Exc L3-4 RORB CUX2	-0.0028	4.96E-01	0.0319	1.08E-01
NLK	Exc L3-4 RORB CUX2	0.0016	5.33E-01	-0.0026	8.41E-01
MAPK8IP2	Exc L3-4 RORB CUX2	0.0034	5.53E-01	0.0740	6.33E-03
WNT4	Exc L3-4 RORB CUX2	-0.0061	5.60E-01	-0.0443	3.97E-01
FZD5	Exc L3-4 RORB CUX2	-0.0043	5.62E-01	-0.0607	1.08E-01
SENP2	Exc L3-4 RORB CUX2	0.0014	5.81E-01	-0.0009	9.44E-01
LGR6	Exc L3-4 RORB CUX2	-0.0064	6.18E-01	-0.0437	3.18E-01
WNT9A	Exc L3-4 RORB CUX2	-0.0036	6.52E-01	-0.0116	7.70E-01
NKD1	Exc L3-4 RORB CUX2	-0.0043	6.64E-01	-0.0228	6.50E-01
CTNNB1	Exc L3-4 RORB CUX2	0.0010	7.11E-01	0.0056	6.79E-01
LGR4	Exc L3-4 RORB CUX2	0.0012	7.17E-01	-0.0218	1.76E-01
DKK3	Exc L3-4 RORB CUX2	-0.0020	7.35E-01	0.0635	1.70E-02
CBY1	Exc L3-4 RORB CUX2	0.0010	7.55E-01	-0.0226	1.74E-01
DUSP16	Exc L3-4 RORB CUX2	-0.0013	7.74E-01	-0.0254	2.41E-01
FZD9	Exc L3-4 RORB CUX2	-0.0023	8.35E-01	0.1081	4.22E-02
STK3	Exc L3-4 RORB CUX2	-0.0008	8.37E-01	-0.0328	8.21E-02
WNT3A	Exc L3-4 RORB CUX2	0.0032	8.38E-01	-0.0566	4.90E-01
MAPK8IP1	Exc L3-4 RORB CUX2	0.0011	8.43E-01	0.0525	5.33E-02
RSPO4	Exc L3-4 RORB CUX2	0.0013	8.70E-01	-0.0512	2.14E-01
ROR2	Exc L3-4 RORB CUX2	0.0012	8.91E-01	-0.0302	4.91E-01
FRAT2	Exc L3-4 RORB CUX2	-0.0012	8.97E-01	0.0814	5.63E-02
FRAT1	Exc L3-4 RORB CUX2	0.0010	9.07E-01	0.1018	1.17E-02
DUSP10	Exc L3-4 RORB CUX2	-0.0014	9.23E-01	-0.0546	4.34E-01
DVL2	Exc L3-4 RORB CUX2	0.0003	9.28E-01	0.0066	6.98E-01
FZD7	Exc L3-4 RORB CUX2	0.0007	9.48E-01	0.1038	4.70E-02
WNT5B	Exc L3-4 RORB CUX2	0.0001	9.83E-01	-0.0132	5.80E-01

Journal of Cell Science • Supplementary information
